# The stigma associated with gestational diabetes mellitus: A scoping review

**DOI:** 10.1016/j.eclinm.2022.101614

**Published:** 2022-08-11

**Authors:** Emma Davidsen, Helle Terkildsen Maindal, Morten Hulvej Rod, Kasper Olesen, Molly Byrne, Peter Damm, Karoline Kragelund Nielsen

**Affiliations:** a Health Promotion Research, Copenhagen University Hospital – Steno Diabetes Center Copenhagen, Borgmester Ib Juuls Vej 83, 2730 Herlev, Denmark; b Department of Public Health, Aarhus University, Bartholins Allé 2, 8000 Aarhus C, Denmark; c National Institute of Public Health, University of Southern Denmark, Studiestræde 6, 1455 Copenhagen K, Denmark; d Health Behaviour Change Research Group, School of Psychology, National University of Ireland, Galway, University Road, Galway, Ireland; e Center for Pregnant Women with Diabetes, Department of Obstetrics, Rigshospitalet, Juliane Maries Vej 8, 2100 Copenhagen Ø, Denmark; f Department of Clinical Medicine, University of Copenhagen, Blegdamsvej 3B, 2200 Copenhagen N, Denmark

**Keywords:** Gestational diabetes mellitus, Stigma, Scoping review

## Abstract

**Background:**

Gestational diabetes mellitus (GDM) affects an increasing number of pregnant women globally. Although studies have identified psychosocial ramifications associated with GDM, stigma in the form of experienced discrimination and self-blame and its consequences have received limited attention. Our objective was to examine the current evidence on stigma, as experienced among women with GDM, including the potential adverse consequences hereof.

**Methods:**

A scoping review was conducted with citations retrieved from the databases MEDLINE, CINAHL, EMBASE and, PsycINFO. Studies published before 15 June 2022, when the search was conducted, were included.

**Findings:**

We identified 1388 citations and included 44 in the review. We found that women with GDM may experience stigma in the form of overt discrimination from healthcare personnel and relatives, and in the form of internalised stigma, such as guilt and shame. Identified consequences include avoidance of screening, not following dietary recommendations nor reporting blood glucose readings, social isolation, and poor mental wellbeing. No estimates of stigma prevalence were identified.

**Interpretation:**

Existing evidence shows that women with GDM report stigma, which may affect both their mental and physical health. Further investigations into the prevalence of stigma and long-term consequences of stigma are much needed.

**Funding:**

The funders of the study had no role in study design, data collection, data analysis, data interpretation, or writing of the report.

**Research in context**

**Evidence before this study**

Research shows that both type 1 and 2 diabetes are encumbered by stigma, but gestational diabetes mellitus (GDM)-specific stigma has not been studied systematically. GDM has been documented to have psychosocial ramifications amongst the affected women, such as feeling guilty or feeling like a failure. These reactions indicate that there may be a stigma associated with having GDM. No previous review has specifically and comprehensively investigated GDM-specific stigma.

**Added value of this study**

This scoping review is the first of its kind to systematically investigate GDM-specific stigma, providing the most comprehensive synthesis of literature investigating overt discrimination, internalised stigma, and adverse consequences associated with GDM-specific stigma. We identified various sources of GDM discrimination, such as healthcare personnel and relatives; several examples of internalised stigma amongst women with GDM, expressed as self-blame and shame; and numerous linked adverse consequences. Finally, although the existence of GDM-specific stigma is well-documented, no studies have investigated the prevalence of either overt discrimination or internalised stigma amongst women with GDM.

**Implications of all the available evidence**

We have identified that GDM may be associated with stigma for some women. Healthcare personnel should consider these findings when providing care to women with GDM and researchers should take GDM-specific stigma into account when designing health interventions targeting women with GDM. To alleviate blame and misunderstandings about GDM from the immediate family, relatives of women with GDM ought to be included in counselling and caring for women with GDM. Finally, GDM-specific stigma needs to be further investigated to enhance our knowledge on the prevalence and associated long-term health consequences.

Alt-text: Unlabelled box

## Introduction

Worldwide, gestational diabetes mellitus (GDM) is estimated to affect more than 17 million live births annually.^1^ GDM is usually a transient form of glucose intolerance diagnosed during pregnancy.^2^ However, women with prior GDM are at high risk of adverse health outcomes, such as type 2 diabetes and cardiovascular disease, and their children are at risk of obesity and insulin resistance through developmental programming.^3^, ^4^, ^5^, ^6^ Thus, GDM is a prime example of a condition of interest in the field of developmental origins of health and disease (DOHaD) focusing on how exposures during early life, including in-utero, influence the risk of later conditions, such as diabetes and cardiovascular diseases.^7^ Growing evidence has emerged from DOHaD on the importance of early life exposures on adult health and disease, but DOHaD also creates new perceptions of where to place responsibility for individual disease-related risks, behaviours and, thereby, potential blame. Specifically, a focus on the imprint of maternal behaviours on foetal health entails a risk that mothers will increasingly be held responsible and blamed for their own and their offspring's health in the short and long term. Researchers have even warned that the findings from the field of DOHaD may be making ‘scapegoats of mothers’.^8^ Consequently, it appears plausible that women with cardiometabolic conditions during pregnancy, such as GDM, may experience assignment of blame and stigmatisation.

In this paper, we conceptualise stigma according to Link & Phelan and Earnshaw & Chaudoir.^9^^,^^10^ According to Link & Phelan, stigma consists of the interrelated components: labelling, stereotyping, separation, status loss and discrimination.^9^ Labelling and stereotyping create a differentiation – or *separation* – between ‘us’ and ‘them’, which may lead to status loss both in society and in the healthcare system. This status loss may in turn also lead to discrimination.^10^ Importantly, the components must coexist in a power situation that allows them to unfold.^9^ Moreover, experienced stigma can be the result of individual and structural discrimination, but it can also be the result of psychosocial mechanisms, where the stigmatised persons’ perceptions of their own stigma influence how they interact with and experience their surroundings.^9^ In other words, as conceptualised by Earnshaw & Chaudoir, stigma can also be internalised when the stigmatising stereotypes and assumptions are absorbed and believed by the stigmatised person herself, resulting in what has been coined self-stigma or internalised stigma.^10^ Research from similar fields have found that 76% of people with type 1 diabetes and 52% of people with type 2 diabetes report diabetes-related stigma, with a higher prevalence amongst female respondents.^11^ GDM-specific stigma, however, has not received the same attention and the current evidence has not been examined in a comprehensive and combined manner.

In this scoping review, we therefore aim to examine the evidence on GDM and stigma, more specifically relating to the following research questions: 1) in which ways and to what extent do women with GDM experience stigmatisation? and 2) what are the potential adverse consequences of GDM-related stigmatisation?

## Methods

We conducted a scoping review, as this design is useful to map the literature on an emerging topic as well as provide insights into avenues for future research.^12^ GDM-specific stigma is a new field of research and we wanted to gain a broad insight into existing literature relating to GDM and stigma. The reporting was guided by the Preferred Reporting Items for Systematic Reviews and Meta-Analyses Scoping Review extension guide (PRISMA-ScR).^13^ The scoping review protocol has been registered in Open Science Framework (https://doi.org/10.17605/OSF.IO/JVG7S).

### Data sources and search strategy

An initial search of PUBMED/MEDLINE was undertaken to identify literature on the topic. Identified keywords and index terms were then used to develop a full systematic search strategy for the databases PUBMED/MEDLINE, CINAHL, EMBASE and PsychINFO (Tables 1 and 2). Open Grey and GreyLit were searched to identify unpublished literature and the reference lists of included studies as well as relevant reviews were screened to detect any additional literature. The final literature search was conducted on the 15^th^ of June 2022.

**Table 1:** Index terms and keywords used for the literature search.

Theme	Search
Gestational diabetes mellitus	Gestational diabetes *or* Gestational diabetes mellitus *or* GDM *or* (Diabetes AND Pregnancy)
*AND*	
Enacted stigma	Stigma *or* Stigmatisation *or* Social stigma *or* Discrimination *or* Prejudice
*OR*	
Internalised stigma	Self-stigma *or* Guilt *or* Blame *or* Self-blame *or* Shame *or* Internalised stigma *or* Fault *or* Contempt *or* Remorse *or* Self-disgust *or* Emotional distress
*OR*	
Experienced GDM care	Care experience *or* Pregnancy care

**Table 2:** Example of full search strategy in PUBMED/MEDLINE.

MEDLINE via PUBMED	
	**Search terms**
1.	“Diabetes, Gestational”[MeSH Terms]
2.	“gestational diabet*”[Title/Abstract] OR gdm [Title/Abstract]
3.	(“Diabetes Mellitus”[Mesh Terms] OR diabet* [Title/Abstract]) AND (“Pregnant Women”[Mesh Terms] OR pregnan* [Title/Abstract])
4.	1 OR 2 OR 3
5.	“Social Stigma”[MeSH Terms] OR “Social Discrimination”[MeSH Terms] OR “Prejudice”[MeSH Terms]
6.	stigma*[Title/Abstract] OR discriminat*[Title/Abstract] OR prejudice*[Title/Abstract]
7.	5 OR 6
8.	“Emotions”[MeSH Terms]
9.	self-stigma*[Title/Abstract] OR selfstigma*[Title/Abstract] OR blame*[Title/Abstract] OR self-blame*[Title/Abstract] OR selfblame*[Title/Abstract] OR guilt*[Title/Abstract] OR fault*[Title/Abstract] OR contempt*[Title/Abstract] OR remorse*[Title/Abstract] OR self-disgust[Title/Abstract] OR selfdisgust[Title/Abstract] OR “emotional distress*”[Title/Abstract]
10.	8 OR 9
11.	"Patient Satisfaction"[MeSH Terms]
12.	“patient satisfaction*”[Title/Abstract] OR “care experience*”[Title/Abstract] OR “pregnancy care*”[Title/Abstract]
13.	11 OR 12
14.	7 OR 10 OR 13
15.	4 AND 14

### Eligibility criteria

Citations were eligible for inclusion if the study population was 1) women with a current or prior GDM diagnosis, 2) relatives of women with a current or prior GDM diagnosis or 3) healthcare personnel working with women diagnosed with GDM. Further, GDM had to be defined as hyperglycaemia with onset or first diagnosis during pregnancy. Variations of the diagnostic criteria for GDM were not of importance in this review. Studies in English, Danish, Swedish, or Norwegian were included in this review. Studies from all dates of publication were included, however, only original research articles were eligible for inclusion. Studies were excluded if they were not specifically related to a GDM diagnosis or did not report on discrimination, stigma, experiences, or feelings in relation to a GDM diagnosis.

### Study selection

All identified citations were imported to the Covidence software, where duplicates were removed.^14^ All citations were double screened by two authors, ED and KKN, first by title and abstract and since by full text. The citations were assessed based on eligibility and exclusion criteria. Any disagreements between the authors were discussed and consensus was reached.

### Quality appraisal

Included citations were assessed using Joanna Briggs Institute's Critical Appraisal Tool (https://jbi.global/critical-appraisal-tools). As the included citations comprised different study designs (qualitative, cross-sectional and mixed-method studies), two different appraisal tools were utilized: Checklist for Qualitative Research and Checklist for Analytical Cross Sectional Studies. To assess mixed-methods citations a combination of the two checklists was used. All citations were assessed by two authors. Any disagreements were discussed and resolved.

### Data extraction and synthesis

A data charting sheet was created in Covidence and tested on five articles by ED and KKN prior to data extraction to ensure compliance.^14^ The data charting sheet was guided by the Joanna Briggs Institute guidelines for conducting a scoping review (see data charting sheet in Supplementary material file 1).^15^ Data was double extracted by ED and KKN.

After completing the data extraction, ED thematically coded data using the software NVivo 12.^16^ For the analysis ED applied an abductive approach, i.e. a combination of theory- and data-driven coding and theme development.^17^ The extrapolated data was guided by the existing conceptualisations of experienced discrimination and internalised stigma, presented by Link & Phelan and Earnshaw & Chaudoir.^9^^,^^10^ Once the data was extrapolated, ED adopted a data-driven approach to coding, which allowed new themes to emerge. Finally, existing conceptualisations of experienced discrimination and internalised stigma were compared to the data-driven codes to identify whether the codes were in line with existing theory and whether new themes had emerged.

### Role of the funding source

The funders of the study had no role in study design, data collection, data analysis, data interpretation, or writing of the report.

## Results

**Figure 1:**
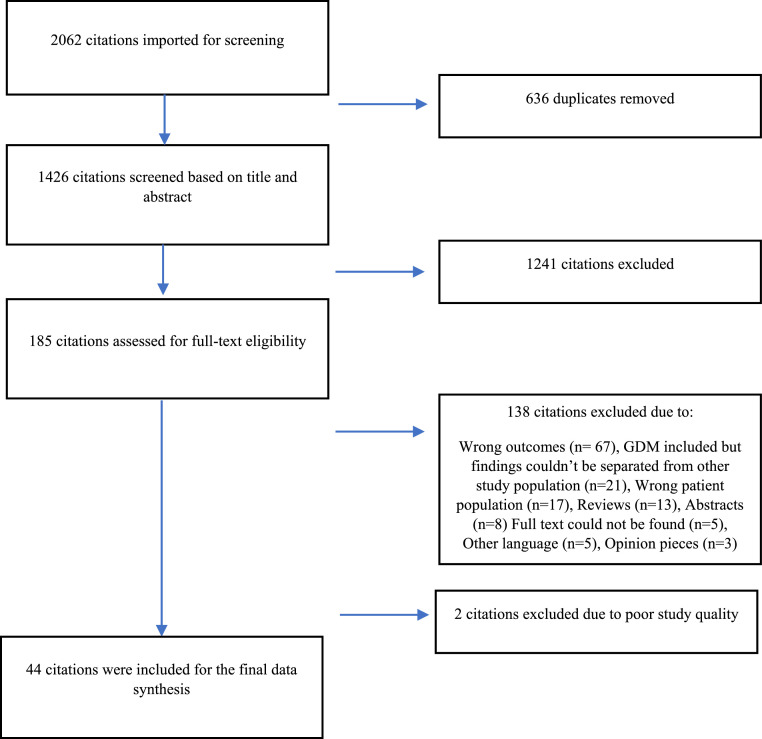
PRISMA flowchart illustrating inclusion and exclusion of citations.

In total, 1388 unique citations were identified in the search; 184 citations were eligible for full text screening, 46 citations were critically appraised, and 44 citations were included in the final analysis (see PRISMA flow chart in Figure 1).

### Study characteristics

Of the included citations, 43 were peer-reviewed articles on original research, and one was a published non-peer-reviewed master's thesis. The earliest citations were from 1994, however, more than 80% of the articles were published after 2012. In total, 879 women with current or prior GDM and 119 healthcare personnel were included. The included citations were from 19 different countries across five continents. The studies were primarily qualitative (*n*=41), with individual interviews and focus group discussions as the dominating methods employed. The remaining three articles comprised one cohort study and two mixed-method studies. Overall, the included citations were of high quality; however, two citations were not included due to a low quality assessment (see supplementary material, appendix b). We did not identify any studies investigating the prevalence of experienced or internalised GDM-specific stigma. The included citations are presented in Table 3.

**Table 3:** Overview of included citations.

Author & year	Country	Study design & data collection method	Study population	Types of identified GDM stigma
Lawson & Rajaram, 1994	United States	Qualitative research; Interviews	17 women diagnosed with GDM	Internalised stigma
Levy-Shiff et al, 2002	Israel	Case-control study; questionnaires and clinical measures	153 pregnant women with singleton offspring. 53 had PGDM*, 51 had GDM and 40 had nondiabetic pregnancy	Internalised stigma
Evans & O'Brien, 2005	Canada	Qualitative research; Interviews	12 women diagnosed with GDM	Experienced discrimination & Internalised stigma
Hjelm et al, 2008	Sweden	Qualitative research; Interviews	23 women diagnosed with GDM	Internalised stigma
Graco et al, 2009	Australia	Qualitative research; Interviews	10 women diagnosed with GDM	Internalised stigma
Doran & Davis, 2010	Tonga	Qualitative research; Interviews	11 women who had developed GDM in the previous 12 months and 10 health professionals	Internalised stigma
Razee et al, 2010	Australia	Qualitative research; Interviews	57 women who had GDM 6-26 months previously	Internalised stigma
Persson et al, 2010	Sweden	Qualitative research; Interviews	10 women diagnosed with GDM	Experienced discrimination & Internalised stigma
Wazqar & Evans, 2012	Canada	Qualitative research; Interviews	12 women diagnosed with GDM	Experienced discrimination & Internalised stigma
Carolan et al, 2012	Australia	Qualitative research; Interviews & focus group discussions	15 women diagnosed with GDM	Internalised stigma
Nielsen et al, 2012	8 low and middle-income countries	Case report; Interviews	10 GDM project managers	Experienced discrimination
Hirst et al, 2012	Vietnam	Qualitative research; Focus group discussions	34 women diagnosed with GDM	Internalised stigma
Abraham & Wilk, 2014	United States	Qualitative research; Interviews	10 women with a history of GDM in the last 2 to 5 years	Experienced discrimination & Internalised stigma
Ghaffari et al, 2014	Iran	Qualitative research; Interviews	25 women diagnosed with GDM	Internalised stigma
Neufeld, 2014	United States	Qualitative research; Interviews & focus group discussions	25 health advisors and 29 women diagnosed with GDM	Experienced discrimination
Hui et al, 2014	United States	Mixed method	30 women diagnosed with GDM	Internalised stigma
Tang et al, 2015	United States	Qualitative research; Interviews	23 women diagnosed with GDM within 12 months of delivery	Internalised stigma
Kilgour 2015	Australia	Qualitative research; Interviews	13 women diagnosed with GDM	Internalised stigma
Burkett et al, 2016^a^	South Africa	Qualitative research; Focus group discussions	16 women diagnosed with GDM	Experienced discrimination
Darroch et al, 2016	Canada	Qualitative research; Interviews & focus group discussions	26 women diagnosed with GDM (1 excluded from analysis)	Experienced discrimination & Internalised stigma
Ge et al, 2016	China	Qualitative research; Interviews	17 women diagnosed with GDM	Experienced discrimination & Internalised stigma
Whitty-Rogers et al, 2016	United States	Qualitative research; Interviews	9 Mikmaq women diagnosed with GDM	Experienced discrimination
Draffin et al, 2016	United Kingdom	Qualitative research; Focus group discussions	19 women currently pregnant with GDM or a history of GDM	Internalised stigma
Gray et al, 2017	United States	Qualitative research; Focus group discussions	16 women diagnosed with GDM	Experienced discrimination & Internalised stigma
Jarvie et al, 2017	United Kingdom	Qualitative research; Interviews	27 women with co-existing BMI >30 and GDM	Experienced discrimination
Carolan-Olah et al, 2017	United States	Qualitative research; Interviews	18 women diagnosed with GDM	Internalised stigma
Stotz et al, 2017	United States	Qualitative research; Interviews & focus group discussions	5 women with a previous diagnosis of GDM and/or T2D during pregnancy	Experienced discrimination & Internalised stigma
Svensson et al, 2018	Denmark	Qualitative research; Interviews	6 women diagnosed with GDM (1 during pregnancy, 5 postpartum)	Internalised stigma
Eades et al, 2018	Scotland, United Kingdom	Qualitative research; Interviews	16 women diagnosed with GDM	Internalised stigma
Siad et al, 2018	Canada	Mixed method	10 women diagnosed with GDM	Experienced discrimination
Parsons et al, 2018	United Kingdom	Qualitative research; Interviews & focus group discussions	50 women diagnosed with GDM	Experienced discrimination & Internalised stigma
McParlin et al, 2019	United Kingdom	Qualitative research; Interviews	12 women diagnosed with GDM	Internalised stigma
Parsons et al, 2019	United Kingdom	Qualitative research; Interviews & focus group discussions	35 women with previous GDM participated in focus groups and 15 women with previous GDM participated in semi-structured interviews	Experienced discrimination & Internalised stigma
Casey et al, 2019	Australia	Qualitative research; Interviews	6 women diagnosed with GDM	Internalised stigma
Kilgour et al, 2019	Australia	Mixed method	13 women diagnosed with GDM, 24 clinicians (interviews); 79 discharge summaries; and 30 GPs and 30 hospital doctors participated in the survey.	Internalised stigma
Harrison et al, 2019	Australia	Qualitative research; Interviews	27 women diagnosed with GDM	Internalised stigma
Dickson et al, 2020	South Africa	Qualitative research; Focus group discussions	10 women diagnosed with GDM	Experienced discrimination & Internalised stigma
Eades et al, 2020	Online – authors from the United Kingdom	Qualitative research; Framework analysis	646 posts in 137 threads from 282 unique users	Experienced discrimination & Internalised stigma
Boyd et al, 2020	England	Qualitative research; Interviews	27 women diagnosed with GDM	Experienced discrimination
Muhwava et al, 2020	South Africa	Qualitative research; Interviews & focus group discussions	35 women diagnosed with GDM	Experienced discrimination & Internalised stigma
Jakobsen et al, 2021	Denmark	Qualitative research; Interviews	9 women with prior GDM	Internalised stigma
Ørtenblad et al, 2021	Denmark	Qualitative research; focus group discussions	32 women with current or prior GDM	Experienced discrimination & Internalised stigma
Sharma et al, 2021	Norway	Qualitative research; focus group discussions	28 women diagnosed with GDM	Internalised stigma
Toft et al, 2021	Norway	Qualitative research; Interviews	14 women diagnosed with GDM	Experienced discrimination & Internalised stigma

a Non-peer reviewed master thesis, *Pre-gestational diabetes mellitus.

### Labelling, stereotyping and separation

The identified literature suggests that women diagnosed with GDM may experience GDM-specific stigma in several ways. Negative labelling and stereotyping have for instance been documented in a study by Burkett et al where healthcare personnel referred to women with GDM as ‘diabetics’, ‘full of drama’ and as someone who would ‘trick’ them and lie to them about their treatment.^18^ In other studies, women with GDM reported that they felt they were being labelled a ‘diabetic’ with an ‘at-risk pregnancy’ as opposed to just being pregnant.^19^^,^^20^ One woman reported ‘I want them to treat me like a human. Treat me, not my diagnosis’.^21^ Studies have also identified various other negative stereotypes associated with the GDM label, such as women with GDM not being able to control their weight; not engaging in physical activity; being lazy; having poor eating habits; lack of willpower and judgement as well as the notion that women diagnosed with GDM have brought the condition upon themselves through their own failings.^18^, ^19^, ^20^^,^^22^, ^23^, ^24^, ^25^, ^26^ The negative labelling and stereotypes mean that women diagnosed with GDM may be vulnerable to being discredited as ‘bad mothers’ or having unhealthy pregnancies and children.^19^^,^^27^ In some cases, labelling and stereotyping was found to overtly result in status loss, for example in settings where women's health was already neglected or their status in the family or society was particularly rooted in childbearing.^26^^,^^28^

### Experienced discrimination

For women diagnosed with GDM the status loss may also result in experienced discrimination. This was primarily reported as direct individual discrimination. Women diagnosed with GDM have described experiencing discrimination from varying sources, including healthcare personnel, spouses and relatives as well as from the local community and society in general.^18^, ^19^, ^20^, ^21^^,^^23^, ^24^, ^25^, ^26^^,^^29^, ^30^, ^31^, ^32^, ^33^, ^34^, ^35^, ^36^, ^37^, ^38^, ^39^, ^40^, ^41^ From healthcare personnel, women diagnosed with GDM report feeling judged; being told ‘horror stories’ about their unborn child's health; being made fun of for weight gain; not being given a choice regarding treatment; feeling threatened, shamed or ‘chastised’ for not meeting glucose targets; being mistrusted regarding whether they follow the diet; and being treated like ‘irresponsible children’.^18^, ^19^, ^20^, ^21^^,^^23^, ^24^, ^25^, ^26^^,^^29^^,^^30^^,^^33^, ^34^, ^35^^,^^38^^,^^39^^,^^42^ Studies have also documented that the women were being accused by their spouses of laziness, and women with GDM have reported feeling like they were under surveillance and scrutiny from their spouse during day-to-day activities, which may be accompanied by being nagged at and judged for what they ate.^30^^,^^31^^,^^33^^,^^34^ The heightened attention to the women's diet was also reported from people in general.^41^ Discrimination from other family members and relatives is particularly evident in studies conducted in smaller communities in rural China and India as well as in Native American populations where women with GDM experienced that especially their mother-in-law blamed them for the diagnosis; accused them of having unhealthy babies due to GDM; were sarcastic in their remarks; assumed the women would have diabetes for the rest of their lives; or even accused them of having kept diabetes a secret before marriage.^32^^,^^40^^,^^43^ Finally, women diagnosed with GDM may also experience discrimination from society and their local communities. Examples include women experiencing that their community ‘talks bad about it [GDM]’; said they were going to die; told them that it was their fault they had GDM; blamed the women for having GDM through multiple pregnancies; said that GDM was ‘false diabetes’ or said GDM was contagious.^18^, ^19^, ^20^, ^21^^,^^36^

### Internalised stigma

In addition, the literature also suggests that women diagnosed with GDM may experience internalised stigma. In fact, internalised stigma was identified as the most reported form of stigma experience in the literature. Thus, numerous studies have documented that women diagnosed with GDM report feeling responsible for their diagnosis and experiencing feelings of guilt, self-blame, failure, embarrassment, sadness, shame and negative self-talk.^20^^,^^22^, ^23^, ^24^, ^25^, ^26^, ^27^, ^28^, ^29^^,^^31^, ^32^, ^33^, ^34^, ^35^, ^36^, ^37^^,^^39^^,^^41^^,^^44^, ^45^, ^46^, ^47^, ^48^, ^49^, ^50^, ^51^, ^52^, ^53^, ^54^, ^55^, ^56^, ^57^ In some studies, these reactions and perceptions were reported to decrease as the women familiarise themselves with the diagnosis and succeed in managing their GDM.^32^ However, in some cases, the feelings were also presented as lasting and impactful.^33^^,^^34^ Feelings of responsibility and guilt were typically related to concerns for the unborn child and studies even suggest that some women diagnosed with GDM feel responsible for previous miscarriages.^22^^,^^56^ Feelings of failure have been reported, especially in relation to ‘failing’ the glucose tolerance test and being diagnosed with GDM, but also in terms of feeling like they failed the unborn child if insulin treatment was required.^20^^,^^26^^,^^28^^,^^33^^,^^37^^,^^39^^,^^41^^,^^53^ In a cohort study, Levy-Schiff and colleagues also found that women with GDM report a higher degree of negative pregnancy-related emotions, such as disappointment, guilt, and worry, than women with no known diabetes during pregnancy.^54^

### Adverse consequences of GDM-related stigmatisation

The consequences of GDM stigma have scarcely been studied. However, from qualitative studies we have evidence of the short-term consequences as reported and experienced by women themselves.

Consequences ascribed to GDM-specific stigma by the women include avoidance of screening/testing, both during and after pregnancy; not reporting on blood glucose readings; disordered eating; not wanting more children; stress; social isolation; loneliness and not prioritising own health after pregnancy.^18^, ^19^, ^20^, ^21^^,^^25^, ^26^, ^27^, ^28^^,^^31^^,^^33^^,^^34^^,^^37^, ^38^, ^39^, ^40^, ^41^^,^^45^^,^^49^^,^^57^^,^^58^, ^59^, ^60^, ^61^ Women diagnosed with GDM have reported negative interactions with healthcare personnel, guilt, fear, and not wanting to identify with diabetes as reasons for not going to scheduled appointments with a doctor, for misreporting in their daily glucose readings and for not attending oral glucose tolerance tests postpartum.^21^^,^^29^^,^^34^^,^^37^ Not attending screening for GDM has further been raised as a possible strategy for pregnant women to not be diagnosed with GDM in the first place and thus avoid stigmatisation.^43^ As a result of feeling like a failure when not meeting their blood glucose targets, some women have reported developing disordered eating, such as starvation or vomiting.^37^^,^^41^^,^^57^^,^^61^ Interestingly, studies indicate that for some women worries, guilt and fear also served as drivers in following the recommended diet and taking insulin during pregnancy.^24^^,^^27^^,^^28^^,^^33^^,^^51^^,^^52^^,^^57^^,^^58^ Yet, due to both experienced discrimination and internalised stigma, some women with GDM have described not disclosing their diagnosis to family and friends, causing them to feel lonely and even isolating themselves from others.^25^^,^^27^^,^^31^^,^^33^^,^^58^ In addition, women with current or prior GDM have also reported feeling guilty or selfish towards their children, if they prioritise their own health instead of spending time with their children.^26^^,^^34^^,^^58^, ^59^, ^60^^,^^62^

### Drivers of stigma

A final theme identified in the literature focuses on some of the potential drivers behind GDM-specific stigma. The internalised stigma, expressed as guilt and self-blame, seems to stem from the large emphasis on personal agency and responsibility imposed on women with GDM. However, researchers have also argued that women's identities are still being rooted in childbearing and motherhood, which is asserted to be a contributing factor of both the internal pressure to succeed as a 'good woman and mother' and the experienced stigma.^27^^,^^29^^,^^35^^,^^44^ Lack of knowledge has also been identified as a driver of stigma, as healthcare personnel, family, and peers as well as women with GDM may not have sufficient knowledge about GDM, which could lead to inadequate counselling.^24^^,^^42^^,^^47^^,^^48^^,^^53^^,^^63^ This means that the women do not get the necessary information about their diagnosis, which can lead to the reported misunderstandings about GDM and result in self-blame regarding their diagnosis or previous miscarriages. ^18^^,^^20^^,^^23^, ^24^, ^25^^,^^27^^,^^28^^,^^33^, ^34^, ^35^, ^36^, ^37^^,^^39^^,^^44^, ^45^, ^46^, ^47^^,^^49^, ^50^, ^51^, ^52^, ^53^, ^54^, ^55^, ^56^, ^57^, ^58^^,^^63^ Studies further suggest that lack of knowledge about the aetiology and risk factors associated with GDM amongst family and peers may also lead to misunderstandings and accusations towards women diagnosed with GDM, leaving the women feeling judged and frightened.^18^^,^^27^^,^^31^^,^^40^ Finally, co-existing stigmatised conditions, such as being overweight and pregnant, simply being a woman, or belonging to an ethnic minority group also appear to interact with the identified consequences associated with experiencing GDM-specific stigma. 18, 19, 20^,^^24^^,^^27^^,^^28^^,^^31^^,^^41^^,^^43^^,^^44^^,^^58^^,^^61^^,^^64^ Thus, women diagnosed with GDM may be more vulnerable and face greater consequences associated with stigma, if they are subject to several stigmatised conditions at the same time.

## Discussion

In this review, we examined the current evidence on GDM and stigma to explore how and to what extent women diagnosed with GDM experience stigma and what the potential consequences could be. Included citations were of high quality, primarily qualitative studies and no citations specifically reported on the prevalence of GDM-specific stigma.

Guided by Link & Phelan's conceptualisations of stigma, all five aspects of stigma were identified: labelling, stereotyping, separation, status loss and discrimination.^9^ In addition, in line with Earnshaw & Chaudoirs conceptualisation of internalised stigma, we also found that the stigma was internalised by women diagnosed with GDM.

Our findings are consistent with findings from similar fields of research, such as stigma associated with having overweight, type 1 or 2 diabetes.^11^^,^^65^ One of the main sources of GDM-specific stigma identified in this review seem to be healthcare personnel. Similar findings have been identified amongst women with overweight or obesity during pregnancy who have reported healthcare personnel as a source of weight stigma.^66^^,^^67^ Family members, friends and society in general were also identified as potentially stigmatising women with GDM. These findings are echoed by Liu and colleagues, who found that the most reported type of diabetes stigma, across both type 1 and 2 diabetes, was the perception that the diagnosis was a result of having a character flaw or due to failure of personal responsibility.^11^ Among people with type 2 diabetes, family and friends were reported to try to make decisions on their behalf regarding which food they were offered.^68^ In a review investigating social stigma associated with diabetes, Schabert et al argue that the policing experienced from family and friends could originate from concerns for the unborn child's health, while blaming the pregnant woman for exposing the unborn child to the associated risks and complications associated with GDM.^68^ We also found that mother-blame was internalised by women with GDM. Internalised stigma has likewise been identified amongst people with overweight and people with type 1 and 2 diabetes.^11^^,^^65^ Thus, a GDM diagnosis potentially creates an interplay between concern and mother-blame, both from others, such as healthcare personnel and relatives, and from the pregnant women themselves.

The identified overlap of different stigmatised conditions affecting women with GDM points to the intersectionality of stigma. The intersectionality of stigma has previously been identified by Hatzenbuehler et al as one of the fundamental causes of health inequalities today, due to the complex and multi-faceted consequences hereof.^69^ Although GDM-specific stigma is relatively understudied and still a new field of research, the parallels that can be drawn between the identified consequences of GDM-specific stigma and those associated with overweight, type 1 and 2 diabetes underline the need to alleviate GDM-specific stigma. The concurrent results also indicate a potential overlap or interaction of stigmas regarding motherhood, weight, and different types of diabetes, which ought to be investigated further.

The identified consequences of GDM-specific stigma in our review resemble existing evidence on stigma in relation to overweight and type 2 diabetes. Studies from these fields have similarly shown that experienced and internalised stigma may have negative effects on mental wellbeing, healthcare seeking behaviours, disordered eating, physical activity and a tendency to social isolation.^11^^,^^65^ Furthermore, the tendency to not disclose a GDM diagnosis to friends and family is in accordance with findings from a study by Nielsen and colleagues where adults with newly diagnosed type 2 diabetes reported not wanting to disclose their diagnosis to others due to shame and fear of stigma.^70^ In this way, the fear of stigma may further contribute to the identified consequences of stigma.

While no studies have examined the impact of experienced GDM-specific stigma on clinical outcomes, findings from similar fields do warrant cause for concern that women diagnosed with GDM, who experience stigma, may face more barriers to engage in healthy behaviours than women who do not experience stigma. Studies within the field of weight stigma have linked experienced weight stigma with increased levels of cortisol and chronic stress, which have been associated with poor cardiometabolic outcomes and premature mortality.^71^ Research amongst people with type 1 diabetes has also linked experienced diabetes stigma with poor glycaemic control and a recent study even linked experienced weight stigma with an increased risk of developing GDM.^72^^,^^73^ Thus, the identified short-term consequences in our study also indicate that there might be a high risk of elevated long-term disease progression among women with GDM who experience GDM-specific stigma.

Based on the findings of this review, we call for healthcare personnel and researchers to consider how they can address and counter-act GDM-specific stigma when providing care for and designing interventions aimed at women with GDM. We also encourage using person-first language, i.e. ‘women with GDM’ instead of ‘GDM women’ or ‘diabetics’ to avoid labelling and inadvertently cause women to feel reduced to their diagnosis.^73^

In addition, it is important that healthcare personnel working with women with GDM have the necessary resources and knowledge to provide satisfactory care. This, amongst other things, includes being trained in addressing sensitive issues without stigmatising, while ensuring that women receive adequate information for reducing complications. As we found in our review, concerns about the health of the unborn child were a strong motivator for the women to follow their treatment; thus, potential adverse consequences for their unborn child should be communicated in a balanced way that allows women to take action, while avoiding self-blame and fear. We suggest involving women with GDM in this task, thus allowing the recipients to contribute to the development of new interventions and information aimed at women with GDM. Finally, we suggest that future interventions targeting women with GDM should address psychosocial and structural factors in GDM treatment, prevention, and foetal health in order not to place unnecessary responsibility on the individual woman thereby inducing blame, shame, and guilt.

While our review identified several studies that touched upon GDM-specific stigma, it is also abundantly clear that future research on the topic is needed. For a start, despite the extensive body of literature with qualitative research reports on GDM-specific stigma, the prevalence of GDM-specific stigma has not been investigated and thus remains unknown. Proper prevalence estimates would not only aid healthcare personnel and researchers in determining the scope of the experienced stigma, but also provide the possibility to investigate whether health promoting interventions or treatment alleviate or increase stigma. In addition, it would be relevant to investigate whether the interaction of a high degree of internalised stigma and the nature of the required treatment, focusing on diet and exercise, effect experiences of blame and self-blame among women with GDM. Furthermore, it is pertinent to investigate possible associations between GDM-specific stigma and clinical outcomes to assess potential long-term consequences. Finally, this review found indications that there is an overlap of stigma amongst women diagnosed with GDM. The interaction of stigmas and consequences related to co-occurring conditions would be relevant to investigate further from a syndemics perspective, to gain a better understanding of the interplay of psychosocial, environmental, and biomedical aspects of GDM in shaping the health and wellbeing of the affected women and children.^74^

To our knowledge, this is the first comprehensive systematic literature review on GDM-specific stigma. We employed no limitations on the years of publications or study design thereby presenting a complete overview of the topic. However, this study is not without limitations. We acknowledge that only including studies in English, Danish, Norwegian, and Swedish may introduce bias, yet only five citations were excluded due to language. Only one of these, a study from Brazil, was considered potentially relevant for the aim of this review. We also acknowledge that the lack of quantitative studies on GDM-specific stigma prevent us from drawing definitive conclusions on the prevalence of stigma or the associated consequences. Furthermore, due to the qualitative nature of the included citations, the identified consequences of GDM-specific stigma are based on the women's experiences and do not allow for quantitative conclusions. This further highlights the need for future research on the topic.

In conclusion, this review shows that women diagnosed with GDM report experiencing stigma, both in terms of overt discrimination from various sources as well as internalised stigma. These findings are in line with existing research from similar research fields, such as overweight in pregnancy and type 1 and 2 diabetes. The identified potential adverse consequences of GDM-specific stigma may affect the women's mental and physical health and some women report not attending screening for GDM to avoid the diagnosis, which should give cause for concern. Our findings call for further research, particularly in terms of measuring the prevalence and investigating the consequences of GDM-specific stigma.

## Contributors

ED and KKN conceived the study idea. ED, HTM, PD, MB, KO and KKN contributed to the study protocol and planned the search. ED conducted the search strategy and performed the literature search. ED and KKN screened citations and extracted the data. ED, KKN, MHR and KO conducted the quality appraisal of included citations. ED analysed and summarised the data and wrote the first draft with contributions from KKN. All authors provided critical revisions to the manuscript and approved the final version.

## Data sharing statement

No individual data has been collected for this study. The data collected and analysed in this review has already been published elsewhere. The extracted data will be made available upon request to the corresponding author. The study protocol was registered in Open Science Framework (DOI: 10.17605/OSF.IO/JVG7S).

## Declaration of interests

ED, KKN, HTM, MHR, and KO are employed at Steno Diabetes Center Copenhagen, a public hospital and research institution under the Capital Region of Denmark, which is partly funded by a grant from the Novo Nordisk Foundation. Funding for ED's PhD was also received from Aarhus University, Denmark. The funders had no role in any part of this study. PD is chair of DSMB for the EMERGE trial: A Randomised Placebo Controlled Trial of the effectiveness of Early MEtformin Care in the Reduction of Gestational Diabetes Mellitus Effects (EMERGE). This is not paid. MB declare no conflict of interests.

## Appendix Supplementary materials

Image, application 1
