# Supplementary material for: The stigma associated with gestational diabetes mellitus: A scoping review
Source: eClinicalMedicine. 2022 Aug 11;52:101614. doi: 10.1016/j.eclinm.2022.101614 (PMC9386490; doi:10.1016/j.eclinm.2022.101614)
Supplement: Supplementary file 1 [file mmc1.docx]

**Appendix B: Critical appraisal of included citations**

|  | **Joanna Briggs Institute’s critical appraisal tool for qualitative studies** | | | | | | | | | | |
| --- | --- | --- | --- | --- | --- | --- | --- | --- | --- | --- | --- |
| **Author, year** | **1. Is there congruity between the stated philosophical perspective and the research methodology?** | **2. Is there congruity between the research methodology and the research question or objectives?** | **3. Is there congruity between the research methodology and the methods used to collect data?** | **4. Is there congruity between the research methodology and the representation and analysis of data?** | **5. Is there congruity between the research methodology and the interpretation of results?** | **6. Is there a statement locating the researcher culturally or theoretically?** | **7. Is the influence of the researcher on the research, and vice- versa, addressed?** | **8. Are participants, and their voices, adequately represented?** | **9. Is the research ethical according to current criteria or, for recent studies, and is there evidence of ethical approval by an appropriate body?** | **10. Do the conclusions drawn in the research report flow from the analysis, or interpretation, of the data?** | **Overall appraisal** |
| Abraham & Wilk, 2014 | Unclear | Yes | Yes | Yes | Yes | No | No | Yes | Yes | Yes | Include |
| Boyd et al, 2020 | Yes | Yes | Yes | Yes | Yes | No | Yes | Yes | Yes | Yes | Include |
| Burkett, 2015 | Yes | Yes | Yes | Yes | Yes | No | No | Yes | Yes | Yes | Include |
| Carolan, 2012 | Yes | Yes | Yes | Yes | Yes | No | No | Yes | Yes | Yes | Include |
| Carolan-Olah, 2017 | Yes | Yes | Yes | Yes | Yes | Yes | No | Yes | Yes | Yes | Include |
| Casey et al, 2018 | Unclear | Yes | Yes | Yes | Yes | Yes | No | Yes | Yes | Yes | Include |
| Darroch & Giles, 2016 | Yes | Yes | Yes | Yes | Yes | Yes | Yes | Yes | Yes | Yes | Include |
| Dickson et al, 2020 | Unclear | Unclear | Yes | Yes | Yes | Yes | No | Yes | Yes | Yes | Include |
| Doran & Davis, 2010 | Unclear | Unclear | Yes | No | Yes | Yes | No | No | Yes | Yes | Include |
| Draffin et al, 2016 | Unclear | Yes | Yes | Unclear | Yes | Yes | No | Yes | Yes | Yes | Include |
| Eades et al, 2020 | Unclear | Yes | Yes | Yes | Yes | No | No | Unclear | Yes | Yes | Include |
| Eades et al, 2018 | Yes | Yes | Yes | Yes | Yes | Unclear | Yes | Yes | Yes | Yes | Include |
| Evans & O'Brien, 2005 | Yes | Yes | Yes | Yes | Yes | Yes | Yes | Yes | Yes | Yes | Include |
| Feighan et al, 2017 | Unclear | Yes | Unclear | Unclear | Unclear | No | No | No | Unclear | Unclear | Exclude |
| Gray et al, 2017 | Unclear | Yes | Yes | Yes | Yes | No | No | Yes | Yes | Yes | Include |
| Ge et al, 2016 | Unclear | Yes | Yes | Yes | Yes | Yes | No | Yes | Yes | Yes | Include |
| Ghaffari et al, 2014 | Unclear | Yes | Yes | Yes | Yes | No | No | Yes | Yes | Yes | Include |
| Graco et al, 2019 | Unclear | Yes | Yes | Yes | Yes | No | No | Yes | Yes | Yes | Include |
| Harrison et al, 2019 | Yes | Yes | Yes | Yes | Yes | Yes | Yes | Yes | Yes | Yes | Include |
| Hirst et al, 2012 | Unclear | Yes | Yes | Yes | Yes | Unclear | Yes | Yes | Yes | Yes | Include |
| Hjelm et al, 2008 | Unclear | Yes | Yes | Yes | Yes | Yes | No | Yes | Yes | Yes | Include |
| Toft et al , 2021 | Unclear | Yes | Yes | Yes | Yes | Yes | Yes | Yes | Yes | Yes | Include |
| Jakobsen et al, 2021 | Yes | Yes | Yes | Yes | Yes | No | Yes | Yes | Yes | Yes | Include |
| Jarvie, 2017 | Unclear | Yes | Yes | Yes | Yes | No | No | Yes | Yes | Yes | Include |
| Kilgour et al, 2015 | Yes | Yes | Yes | Yes | Yes | Yes | Unclear | Yes | Yes | Yes | Include |
| Lawson & Rajaram, 1994 | Unclear | Yes | Yes | Unclear | Yes | No | No | Yes | Yes | Yes | Include |
| McParlin et al, 2019 | Yes | Yes | Yes | Yes | Yes | Yes | No | Yes | Yes | Yes | Include |
| Muhwava et al, 2020 | Unclear | Yes | Yes | Yes | Yes | Yes | No | Yes | Yes | Yes | Include |
| Nielsen et al, 2012 | Unclear | Yes | Yes | Yes | Yes | No | No | Yes | Yes | Yes | Include |
| Ørtenblad et al , 2021 | Unclear | Yes | Yes | Yes | Yes | No | No | Yes | Yes | Yes | Include |
| Parsons et al, 2018 | Unclear | Yes | Yes | Yes | Yes | No | Yes | Yes | Yes | Yes | Include |
| Parsons et al, 2019 | Unclear | Yes | Yes | Yes | Yes | No | No | Yes | Yes | Yes | Include |
| Persson et al, 2010 | Yes | Yes | Yes | Yes | Yes | Yes | Yes | Yes | Yes | Yes | Include |
| Razee et al, 2010 | Unclear | Yes | Yes | Yes | Yes | No | No | Yes | Yes | Yes | Include |
| Sharma et al, 2021 | Yes | Yes | Yes | Yes | Yes | Yes | Yes | Yes | Yes | Yes | Include |
| Siad et al, 2018 | Unclear | Yes | Yes | Yes | Yes | Yes | No | Yes | Yes | Unclear | Include |
| Stotz et al, 2017 | Unclear | Yes | Yes | Yes | Yes | No | No | Yes | Yes | Yes | Include |
| Svensson et al, 2018 | Unclear | Yes | Yes | Yes | Yes | No | No | Yes | Yes | Yes | Include |
| Neufeld , 2014 | Yes | Yes | Yes | Yes | Yes | No | No | Yes | Yes | Yes | Include |
| Tang et al, 2015 | Yes | Yes | Yes | Yes | Yes | Unclear | No | Yes | Yes | Yes | Include |
| Wazqar & Evans, 2012 | Unclear | Yes | Yes | Yes | Yes | No | No | Yes | Yes | Yes | Include |
| Whitty-Rogers et al, 2016 | Yes | Yes | Yes | Yes | Yes | Yes | Yes | Yes | Yes | Yes | Include |
| Hui et al, 2014* | Unclear | Yes | Yes | Yes | Yes | No | No | Yes | Yes | Yes | Include |
| Kilgour et al, 2018* | Unclear | Yes | Yes | Unclear | Yes | No | No | Unclear | Yes | Yes | Include |
| **Joanna Briggs Institute’s critical appraisal tool for analytical cross sectional studies** | | | | | | | | | |  |  |
| **Author, year** | **1. Were the criteria for inclusion in the sample clearly defined?** | **2. Were the study subjects and the setting described in detail?** | **3. Was the exposure measured in a valid and reliable way?** | **4. Were objective, standard criteria used for measurement of the condition?** | **5. Were confounding factors identified?** | **6. Were strategies to deal with confounding factors stated?** | **7. Were the outcomes measured in a valid and reliable way?** | **8. Was appropriate statistical analysis used?** | **Overall appraisal** |  |  |
| Hussain et al, 2014 | Yes | Yes | Not applicable | Not applicable | No | No | Not applicable | No | Exclude |  |  |
| Levy-Shiff et al, 2002 | Yes | Yes | Yes | Yes | Unclear | Unclear | Yes | Yes | Include |  |  |
| Hui et al, 2014* | Yes | Yes | Yes | Yes | Not applicable | Not applicable | Yes | Yes | Include |  |  |
| Kilgour et al, 2018* | No | No | Yes | Yes | No | No | Yes | Yes | Include |  |  |

*Mixed method research. The studies employing mixed methods were assessed using both the qualitative and the cross sectional JBI critical appraisal tools. The overall appraisal was based on the assessment from both checklists.
